# Long non-coding RNA Lethe regulates hyperglycemia-induced reactive oxygen species production in macrophages

**DOI:** 10.1371/journal.pone.0177453

**Published:** 2017-05-11

**Authors:** Carlos Zgheib, Maggie M. Hodges, Junyi Hu, Kenneth W. Liechty, Junwang Xu

**Affiliations:** 1 Department of Surgery, University of Colorado Anschutz Medical Campus, Aurora, Colorado, United States of America; 2 Department of Surgery, Division of Pediatric Surgery, Children’s Hospital Colorado, Aurora, Colorado, United States of America; Cedars-Sinai Medical Center, UNITED STATES

## Abstract

Type 2 diabetes mellitus is a complex, systemic metabolic disease characterized by insulin resistance and resulting hyperglycemia, which is associated with impaired wound healing. The clinical complications associated with hyperglycemia are attributed, in part, to the increased production of reactive oxygen species (ROS). Recent studies revealed that long non-coding RNAs (lncRNAs) play important regulatory roles in many biological processes. Specifically, lncRNA Lethe has been described as exhibiting an anti-inflammatory effect by binding to the p65 subunit of NFκB and blocking its binding to DNA and the subsequent activation of downstream genes. We therefore hypothesize that dysregulation of Lethe’s expression plays a role in hyperglycemia-induced ROS production. To test our hypothesis, we treated RAW264.7 macrophages with low glucose (5 mM) or high glucose (25 mM) for 24h. High glucose conditions significantly induced ROS production and NOX2 gene expression in RAW cells, while significantly decreasing Lethe gene expression. Overexpression of Lethe in RAW cells eliminated the increased ROS production induced by high glucose conditions, while also attenuating the upregulation of NOX2 expression. Similar results was found also in non-diabetic and diabetic primary macrophage, bone marrow derived macrophage (BMM). Furthermore, overexpression of Lethe in RAW cells treated with high glucose significantly reduced the translocation of p65-NFkB to the nucleus, which resulted in decreased NOX2 expression and ROS production. Interestingly, these findings are consistent with the decreased Lethe gene expression and increased NOX2 gene expression observed in a mouse model of diabetic wound healing. These findings provide the first evidence that lncRNA Lethe is involved in the regulation of ROS production in macrophages through modulation of NOX2 gene expression via NFκB signaling. Moreover, this is the first report to describe a role of lncRNAs, in particular Lethe, in impaired diabetic wound healing. Further studies are warranted to determine if correction of Lethe expression in diabetic wounds could improve healing.

## Introduction

The worldwide prevalence of type 2 diabetes mellitus (T2DM) has reached pandemic proportions, affecting millions of people in the U.S. and worldwide. In the US, diabetes is one of the leading causes of morbidity and mortality and is responsible for more than $175 billion in annual healthcare expenditure in the US alone [1]. Diabetes is associated with many complications, including neuropathy, retinopathy, nephropathy, as well as the development of chronic diabetic wounds. Left untreated, diabetic wounds exhibit impaired wound healing, thereby increasing the risk of wound infection and often necessitating toe, foot or leg amputation. In 2014, over 70,000 lower extremity amputations were performed in diabetic patients, and nearly 2/3 of these non-traumatic amputations were preceded by a diabetic wound [2]. The burden of disease attributable to non-healing diabetic wounds represents a significant clinical and economic burden; with the prevalence of type 2 diabetes projected to increase worldwide, the need for effective wound care therapies cannot be understated [3].

Diabetic wounds do not follow the well-orchestrated, progressive course of healing demonstrated by normal wounds [4]. Instead, diabetic wounds demonstrate impaired wound healing do to many factors, including decreased production of growth factors, impaired angiogenesis, and chronic inflammation [5]. In addition, several recent studies have demonstrated that oxidative stress induced by hyperglycemia is a central regulator of the wound healing response in diabetic wounds [6, 7]. Controlled levels of ROS are essential for the elimination of invading bacteria and pathogens. Moreover, low levels of ROS are also vital for other processes important to proper wound healing, such as angiogenesis [8]. However, high levels of ROS can be harmful to the healing process. T2DM is associated with high levels of oxidative stress, resulting in cell damage or death due to increased protein oxidation and lipid peroxidation [9]. In particular, NADP oxidase (NOX) is an enzyme known to produce significant amounts of ROS in wounds, while also being highly expressed by inflammatory cells [10, 11]. Other studies have shown that high levels of ROS, such as H_2_O_2_ and superoxide, are present during the inflammatory phase of wound healing in normal wounds [8, 12]. Arya *et al*. showed that ROS are significantly upregulated in chronic, non-healing diabetic wounds, furthermore, they showed that a relationship exists between oxidative stress and apoptotic markers in lymphocytes of diabetic patients with chronic, non-healing wounds [5]. In diabetic wounds, increased levels of ROS initiate lymphocyte apoptosis, which results in the upregulation of pro-apoptotic proteins and downregulation of anti-apoptotic proteins [13, 14]. Conversely, antioxidants like glutathione (GSH) and ROS scavengers such as superoxide dismutase (SOD) and catalase are significantly downregulated in diabetic wounds [8, 15–18].

In recent years, numerous small, non-coding proteins have been identified to be associated with disease, including the identification of thousands of long non-coding RNAs lncRNAs. This suggests that dysregulated expression or function of lncRNAs might be pathogenic [19]. Until now, the role of lncRNA in diabetic wounds has been largely undefined. Lethe, a long non-coding RNA named after the “river of forgetfulness” in Greek mythology, is induced by pro-inflammatory cytokines via either NFκB or glucocorticoid receptor agonists, functioning in a negative feedback loop to inhibit NFκB-induced signaling and inflammation. Rapicavoli *et al*. showed that Lethe inhibits NFκB by binding to the RelA subunit and blocking its DNA binding and downstream pro-inflammatory gene activation [20]. With the knowledge that the p65-NFkB complex can induce the expression of NOX2, and that NFκB and oxidative stress are known to be induced by inflammatory cells in diabetic wounds, it is suggested that Lethe could play a key role in the regulation of these processes [21].

Thus, we propose the novel hypothesis that hyperglycemia-induced upregulation of oxidative stress is due to the dysregulation of Lethe expression. We also investigate the mechanism by which Lethe may regulate NOX2 expression through the NFκB pathway.

## Materials and methods

### Cell culture and reagents

RAW 264.7 macrophages (ATCC, USA) were maintained in DMEM (Gibco, USA) supplemented with 10% (*v*/*v*) FBS (Gibco, USA), 100μg/ml streptomycin, and 100U/ml penicillin, and incubated at 37°C and an atmosphere of 5% CO2. Cells were serum starved overnight in DMEM with 1% FBS and 100μg/ml streptomycin, and 100U/ml penicillin before being treated with 5mM D-glucose (LG) DMEM, 25mM D-Glucose (HG), 5mM L-glucose, or 25mM L-Glucose for 24 hours according to [22]. RAW in this study was analyzed at passage 2 to 3.

### BMM isolation and flow cytometry analysis

Bone marrow-derived macrophages (BMM) were isolated from the bone marrow of non-diabetic and diabetic mice by flushing the femurs with sterile PBS solution (1X). These cells were cultured and expanded in L929-conditioned medium supplemented with macrophage colony-stimulating factor (M-CSF). Non-Diabetic and diabetic BMM were analyzed at passage 2 to 3. Flow cytometry analysis was done according to [23].

### Animal studies

All animal experiments were approved by the Institutional Animal Care and Use Committee at the University of Colorado Denver—Anschutz Medical Campus and followed the guidelines described in the NIH Guide for the Care and Use of Laboratory Animals. 10 week old age-matched, female, genetically diabetic C57BKS.Cg-m/Leprdb/J mice and heterozygous, non-diabetic, female controls were used in these experiments.

### Mouse wounding protocol

Mice were anesthetized with inhaled isofluorane. Each mouse was shaved and depilated before wounding. The dorsal skin was swabbed with alcohol and Betadine (Purdue Pharma, Stamford, CT). Each mouse underwent a single, dorsal, full-thickness wound (including panniculus carnosum) with an 8-mm punch biopsy (Miltex Inc, York, PA). All wounds were dressed with tegaderm (3M, St Paul, MN), which was subsequently removed on postoperative day 2. Postoperatively, the mice received a subcutaneous injection of an analgesic, Banamine (Schering-Plough Animal Health Corp., Union, NJ). A full-thickness skin sample, centered on the wound, was harvested 1, 3, and 7 days after surgery (n = 5 per timepoint).

### Real time quantitative PCR

Total RNA was extracted with TRIzol reagent (Invitrogen, Carlsbad, CA, USA) according to the manufacturer's established protocol. RNA was converted into cDNA using the SuperScript First-Strand Synthesis System (Invitrogen, Life Technologies). Primers for Lethe, as well regulators of ROS production, such as Nox2, Sod2, Sod3 and catalase, were amplified using the TaqMan gene expression assay (Applied Biosystems). Internal normalization was achieved by using the 18s housekeeping gene. Samples (n = 5 per group) were amplified in triplicate and results were averaged for each individual sample. The ΔΔCT method was used to calculate relative gene expression. Results are reported as mean ± SD.

### Lethe overexpression and transfection

To overexpress Lethe, the pcDNA-Lethe plasmid was constructed by GeneCopoeia (Rockville, MD). The Lethe gene was amplified by a primer designed according to the murine Lethe sequence (NR_036572–1). The empty pcDNA3.1 vector was used as a control. In 6 well culture plates, the RAW or BMM cells were transfected with 2 μg of either pcDNA-Lethe or empty pcDNA3.1 using Lipofectamine 2000 (Invitrogen Life Technologies). After 2h transfection for RAW cells, or 6h transfection for BMM, the medium was replaced with fresh DMEM supplemented with 10% (*v*/*v*) FBS, 100μg/ml streptomycin, and 100U/ml penicillin. 24h following transfection, the cells were processed for ROS or gene expression analysis.

### Intracellular ROS measurement

After the RAW cells reached 70% confluence, they were subjected to transfection before being exposed for 24 hours to normal and high glucose conditions in an FBS-free medium. Intracellular production of hydroxyl, peroxyl, and other ROS was measured by the Cellular Reactive Oxygen Species Detection Assay Kit (Abcam, USA). For BMM cells, ROS was measured for non-diabetic and diabetic BMM at baseline and after transfection. After 24hrs of incubation in high or low glucose conditions, the RAW cells were exposed to 2′,7′-dichlorofluorescein diacetate (DCFDA) for 20 min. The level of intracellular ROS was assessed by the fluorescence emitted by DCFDA after conversion to 2′,7′-dichlorofluorescein by reaction with ROS. The excitation and emission wavelengths were 492 and 521 nm respectively; ROS levels were recorded by arbitrary unit.

### Western blot analysis

Cell lysates were prepared in standard NP-40 lysis buffer (Abcam, USA) supplemented with proteinase and phosphatase inhibitors. Protein lysates were then quantified using the Pierce BCA protein assay kit (Thermo Fisher Scientific, USA). Equal masses of total protein were separated on 4–12% SDS-polyacrylamide mini-gels and blotted onto PVDF membrane (Millipore, USA). Membranes were subsequently blocked, incubated with primary antibodies, and incubated with secondary antibodies according to WesternBreeze Chromogenic Kit (Thermo Scientific, USA). Alkaline phosphatase was detected on the PVDF membranes using a ready-to-use BCIP/NBT substrate (Thermo Scientific, USA) for ready visualization of enzyme-linked antibodies. Rabbit anti-p65, anti-phospho-p65, anti-GAPDH, and anti-TBP antibodies were obtained from Cell Signaling (USA) or Abcam (USA).

### Statistical analysis

Results are expressed as mean ± SD for n = 3 to 5 number of independent experiments. Statistically significant differences in gene expression or ROS production between two groups was assessed by Student T-test. P<0.05 was considered to be statistically significant.

## Results

### Hyperglycemia induces the expression of NOX2 and increases ROS production in macrophages

**Fig 1:**
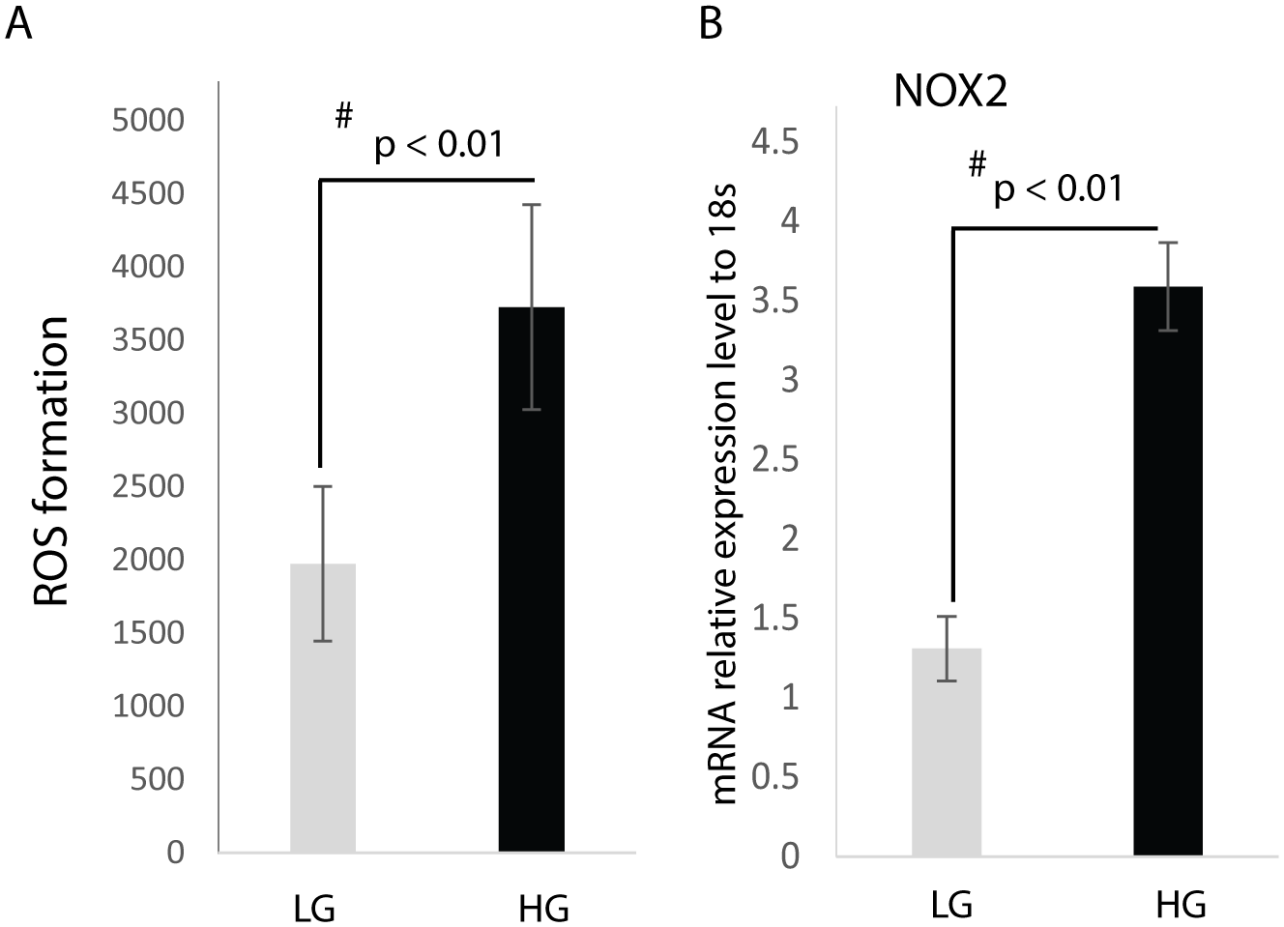
Hyperglycemia induces ROS production and NOX2 gene expression. Raw cells were treated for 24h with: 5 mM D-glucose (LG), or 25 mM D-glucose (HG). (A) ROS levels were measured using the Cellular Reactive Oxygen Species Detection Assay Kit. The data is represented as Mean ± SD, n = 5 per treatment group. (B) RT-qPCR analysis of NOX2 gene expression. Results show increased NOX2 gene expression under high glucose conditions.

After culture of RAW cells in low (5 mM) or high (25 mM) glucose media for 24 hours, our results show that culture of RAW cells in high glucose media significantly induces the production of ROS when compared to RAW cells cultured in low glucose media (Fig 1A).

Following the same experimental protocol as above, we analyzed the effect of hyperglycemia on NOX2 expression. Fig 1B demonstrates that RAW cells express NOX2 when cultured in low glucose media; however, culture in high glucose media resulted in a doubling of NOX2 relative gene expression.

#### Lethe expression is downregulated under hyperglycemic conditions

**Fig 2:**
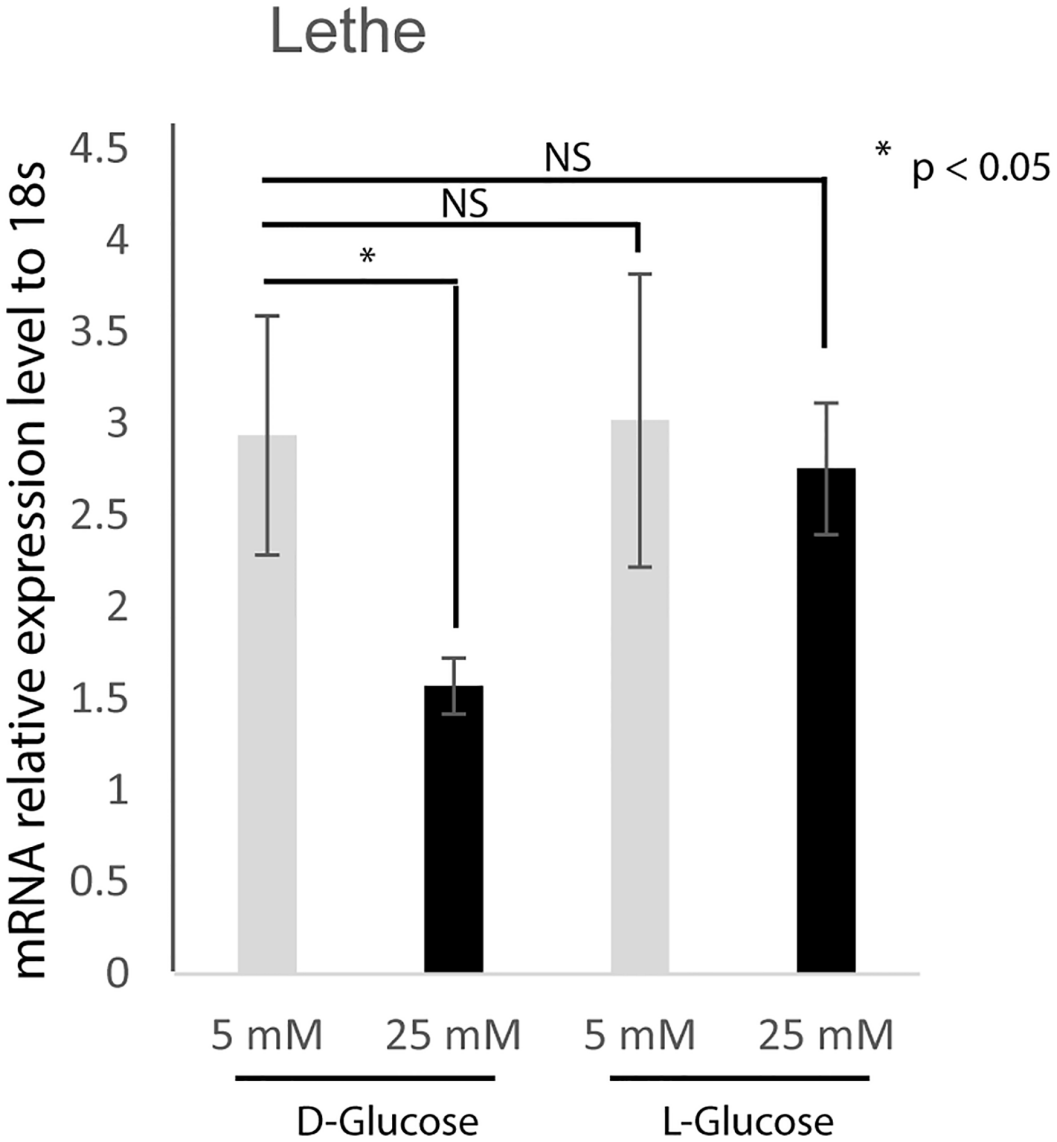
Hyperglycemia reduces Lethe gene expression. RNA analyses by RT-qPCR (mean ± SD, n = 5 per group) showed significantly decreased Lethe gene expression in RAW cells under high glucose conditions (25 mM D-glucose). However, no difference in Lethe gene expression was observed between the L-glucose treated groups compared to the low D-glucose (LG) treated groups.

We subsequently wanted to know whether macrophages express Lethe, and if Lethe gene expression is affected by high glucose conditions. In order to evaluate the response of Lethe gene expression to low glucose and high glucose conditions, RAW cells were cultured in either low or high glucose media or equimolar L-glucose control, and the expression of Lethe was measured by real time PCR. Fig 2 shows that following culture in high glucose media, Lethe gene expression was significantly downregulated when compared to Lethe gene expression in RAW cells cultured in low glucose media. There was no significant difference in Lethe gene expression in RAW macrophages treated with low L-glucose compared to RAW macrophages treated with low D-glucose, indicating that high D-glucose is responsible for down-regulation of Lethe expression.

### Overexpression of Lethe decreases ROS production in RAW macrophages

**Fig 3:**
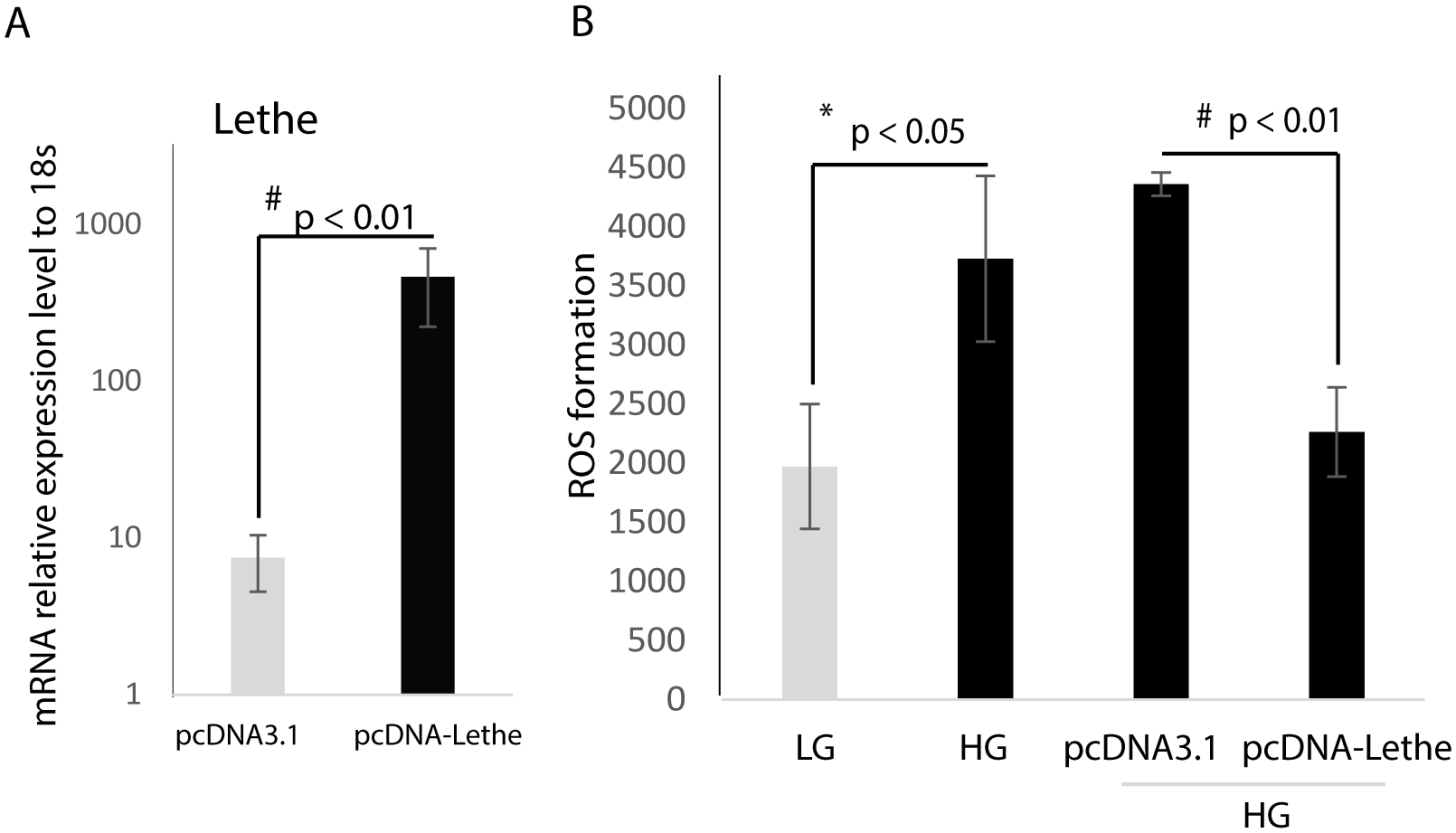
Overexpression of Lethe in RAW macrophage cells decreases ROS production. (A) Overexpression of Lethe gene expression was achieved by plasmid transfection and confirmed by RNA analyses by RT-qPCR (mean ± SD, n = 3 per group), Lethe gene expression was significantly induced in the RAW macrophages transfected with pcDNA-Lethe compared to the RAW macrophages transfected with pcDNA3.1. (B) Under HG condition, ROS production was prevented following transfection of RAW macrophages with pcDNA-Lethe (mean ± SD, n = 5 per group). ROS levels were measured by the Cellular Reactive Oxygen Species Detection Kit.

In order to establish the role Lethe may play in the regulation of ROS production in macrophages, RAW macrophages were transfected with a pcDNA expressing either Lethe or pcDNA3.1, as a control. Our results show that Lethe gene expression is increased in the pcDNA-Lethe-transfected cells, which confirms successful transfection of the RAW cells with pcDNA-Lethe (Fig 3A). Next, we measured ROS production under both high glucose and low glucose conditions, following transfection with either pcDNA-Lethe or pcDNA3.1. Fig 3B shows that Lethe overexpression reduced the formation of ROS in RAW macrophages cultured in high glucose media (high glucose + pcDNA-Lethe) compared to ROS production in RAW macrophages treated the empty vector control that were cultured in high glucose media (high glucose + pcDNA3.1).

### Overexpression of Lethe decreases ROS production in diabetic primary macrophage

**Fig 4:**
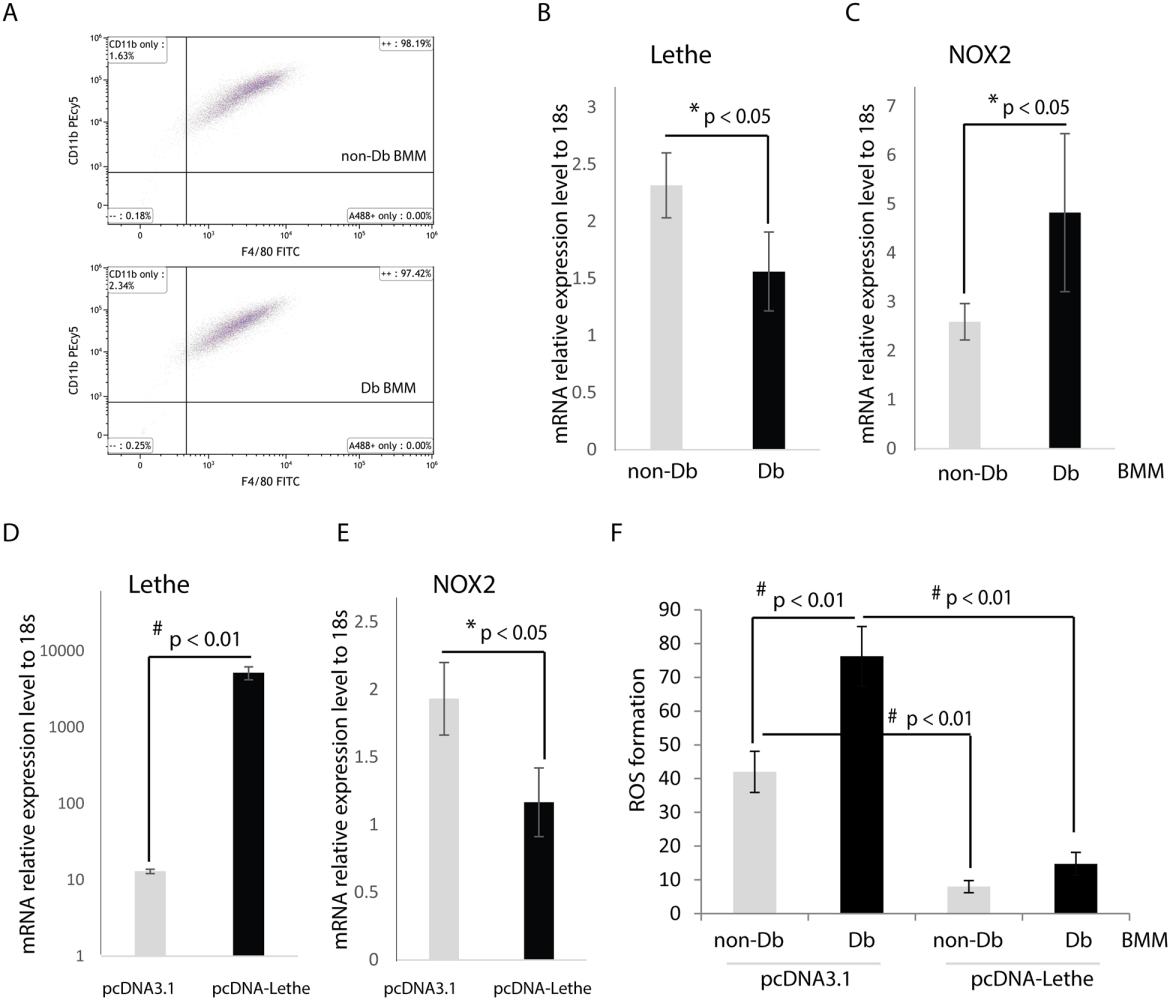
Overexpression of Lethe decreases ROS production in BMM. (A)BMM was confirmed as macrophage by Flow cytometry analysis. Non-diabetic and diabetic BMM showed more than 97% F4/80 and CD11b double positive. (B) Lethe expression was measured by RT-qPCR (n = 3), and Lethe was significantly higher in non-diabetic BMM (p < 0.05) at baseline. (C) NOX2 gene expression was significantly higher in diabetic BMM at baseline (n = 3, p < 0.05), inversely associated with Lethe expression. (D) Lethe overexpression was achieved by pcDNA-Lethe transfection in BMM compared to pcDNA3.1 transfection as control (n = 3, p<0.01). (E) Lethe overexpression significantly reduced NOX2 expression in BMM (n = 3, p<0.05). (F) Diabetic BMM had significantly higher ROS production in the group transfected with the control plasmid pcDNA3.1, compared to the group treated with pcDNA-Lethe. In pcDNA-Lethe transfected groups, both non-Db and Db BMM had significantly lower ROS production compared to the pcDNA3.1 transfected controls (the data are mean ± SD, n = 5 per group).

To better evaluate these findings in a diabetic model, we evaluated the effects of Lethe overexpression in macrophages isolated from the bone marrow (BMM) of non-diabetic and diabetic mice. The identities of BMM was confirmed by Flow Cytometry analysis. In both non-diabetic and diabetic BMM, over 97% cells were double positive for surface antigen F4/80 and CD 11b, indicating they were macrophage (Fig 4A). We measured Lethe expression in BMM, Lethe was significantly higher in non-diabetic BMM compared to diabetic BMM (Fig 4B); while NOX2 was significantly higher in diabetic BMM compared to non-diabetic BMM (Fig 4C). Then we asked whether Lethe played similar role as in RAW macrophage on regulation NOX2 expression in BMM. Fig 4D indicates that Lethe overexpression was achieved by plasmid transfection, Fig 4E shows that overexpression of Lethe was able to significantly down-regulate NOX2 gene expression in BMM. Fig 4F shows that ROS production was significantly higher in diabetic BMM, and overexpression of Lethe significantly attenuated ROS production in non-diabetic and diabetic BMM.

### Overexpression of Lethe reduced NOX2 expression

**Fig 5:**
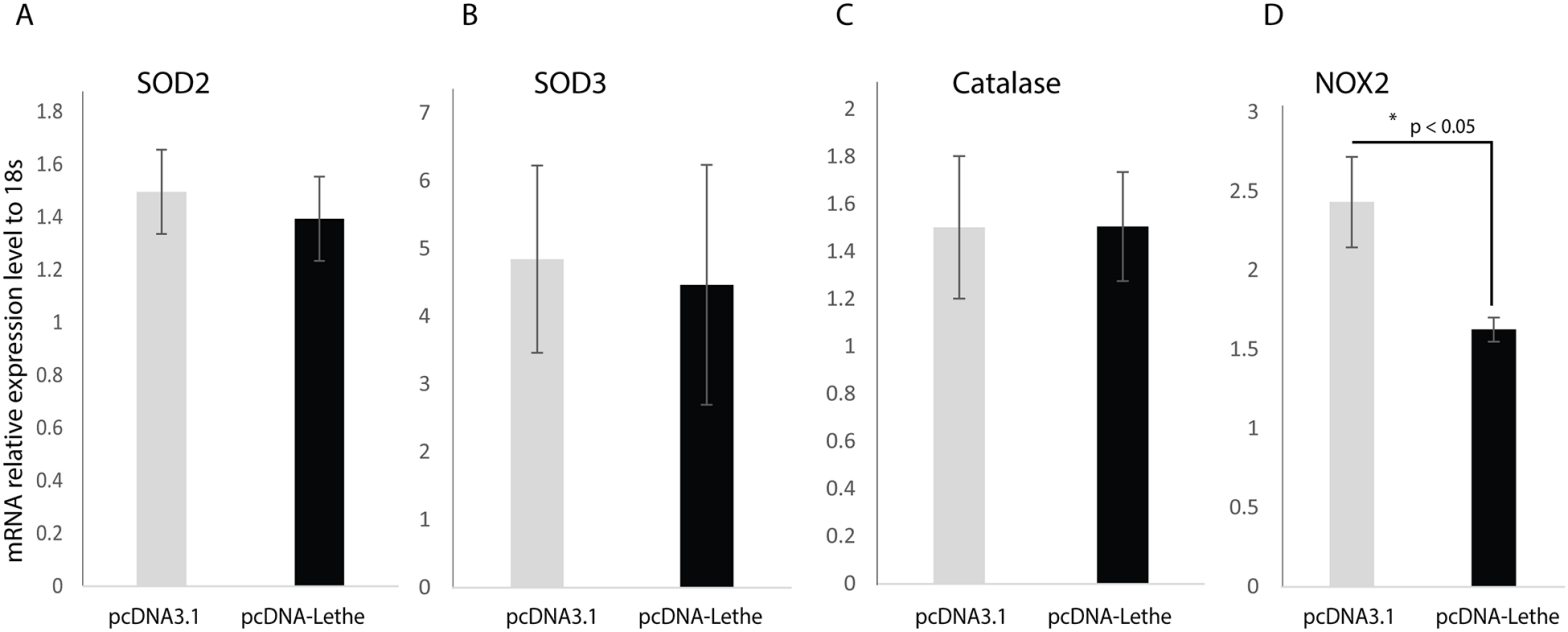
Lethe overexpression reduces NOX2 gene expression. RNA analyses by RT-qPCR (mean ± SD, n = 5 per group) showed no significant change in SOD2 (A), SOD3 (B), and Catalase (C) gene expression, while NOX2 (D) gene expression was significantly decreased following overexpression of Lethe in the RAW macrophages transfected with pcDNA-Lethe.

With a desire to better understand the mechanism by which Lethe overexpression reduced ROS formation in RAW cells cultured under high glucose conditions, we assessed the impact of Lethe overexpression on the gene expression of known ROS scavengers, including NOX2, SOD2, SOD3, and catalase. As shown in Fig 5, our results demonstrate that overexpression of Lethe did not have a significant impact on the gene expression of SOD2, SOD3, or Catalase. However, NOX2 expression was significantly downregulated by Lethe overexpression in RAW cells cultured in high glucose media. These results indicate that the reduction in ROS production due to overexpression of Lethe is mediated by NOX2.

### Lethe regulates NOX2 expression via a p65-NFκB-dependent mechanism

**Fig 6:**
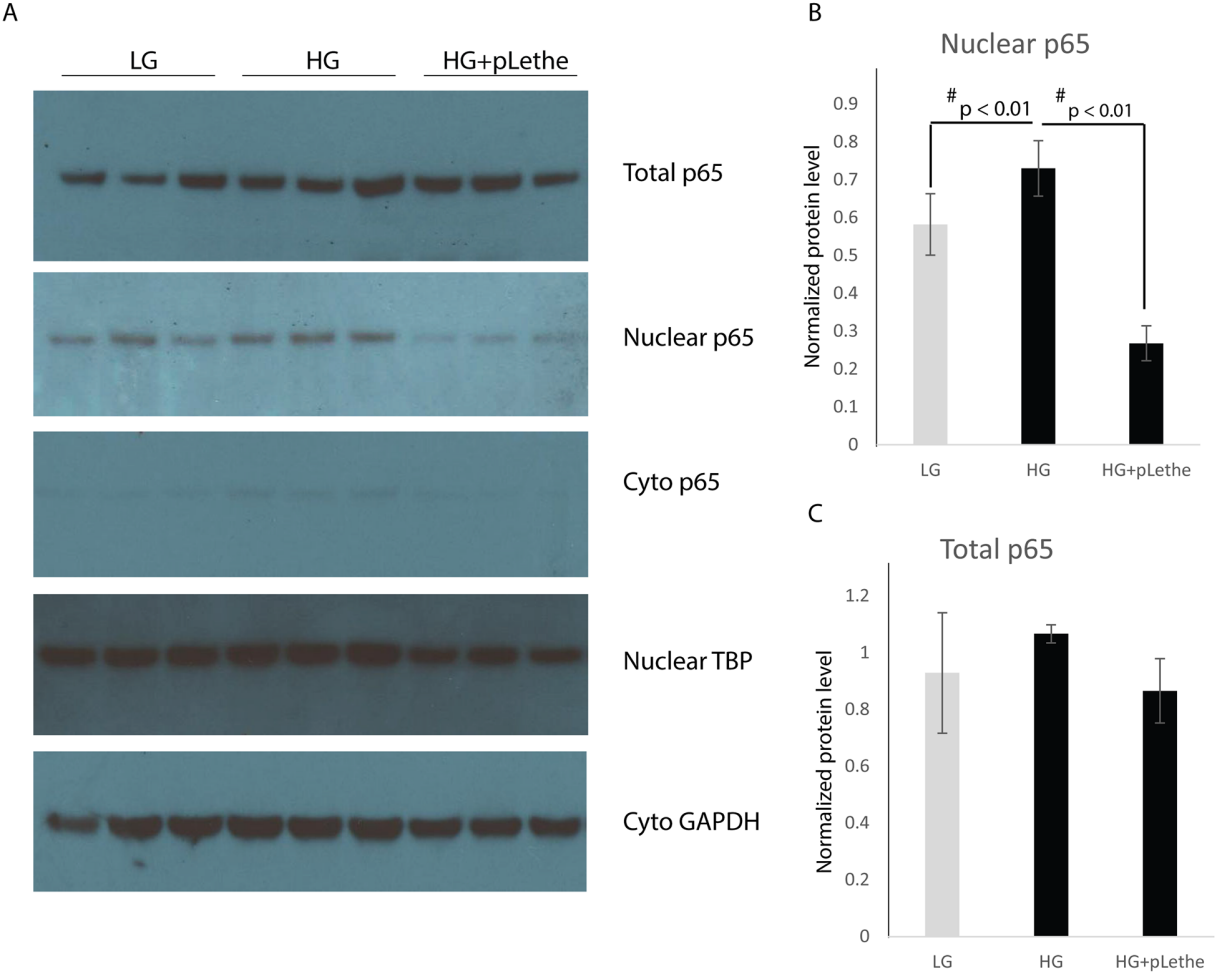
Lethe overexpression prevents translocation of p65 to nucleus. (A) Western blot analysis showed that hyperglycemia induced nuclear p65, while overexpression of Lethe significantly reduced nuclear p65. For protein in nuclear fraction, we used TATA binding protein (TBP) as loading control; for protein in cytoplasmic fraction, we used GADPH as loading control. (B) Protein levels were normalized to cytoplasmic GAPDH and nuclear TBP by the software ImageJ. Results indicated that there was no change in total p65 protein level, while Lethe overexpression significantly reduced nuclear p65 (mean ± SD, n = 3 per group), while no significantly difference in total p65 protein levels between groups.

NFκB has been implicated in the regulation of NOX2 gene expression in various cells, including macrophages [24]. Lethe negatively regulates NFκB signaling by interacting with the p65-NFκB subunit and inhibiting its DNA binding and target gene activation [20]. Thus, we hypothesized that Lethe could be inhibiting NOX2 expression by binding to and inhibiting the translocation of p65-NFκB to the nucleus. Using subcellular fractionation and western blot analysis, we observed that the p65-NFκB protein expression was significantly higher in both nuclear and cytoplasmic fractions in cells cultured in high glucose media (Fig 6A). While total p65-NFκB protein levels were not changed across all the treatment groups, p65-NFκB protein expression was decreased in the nuclear fraction following the overexpression of Lethe (Fig 6B and 6C).

### Lethe expression is decreased in diabetic wounds

**Fig 7:**
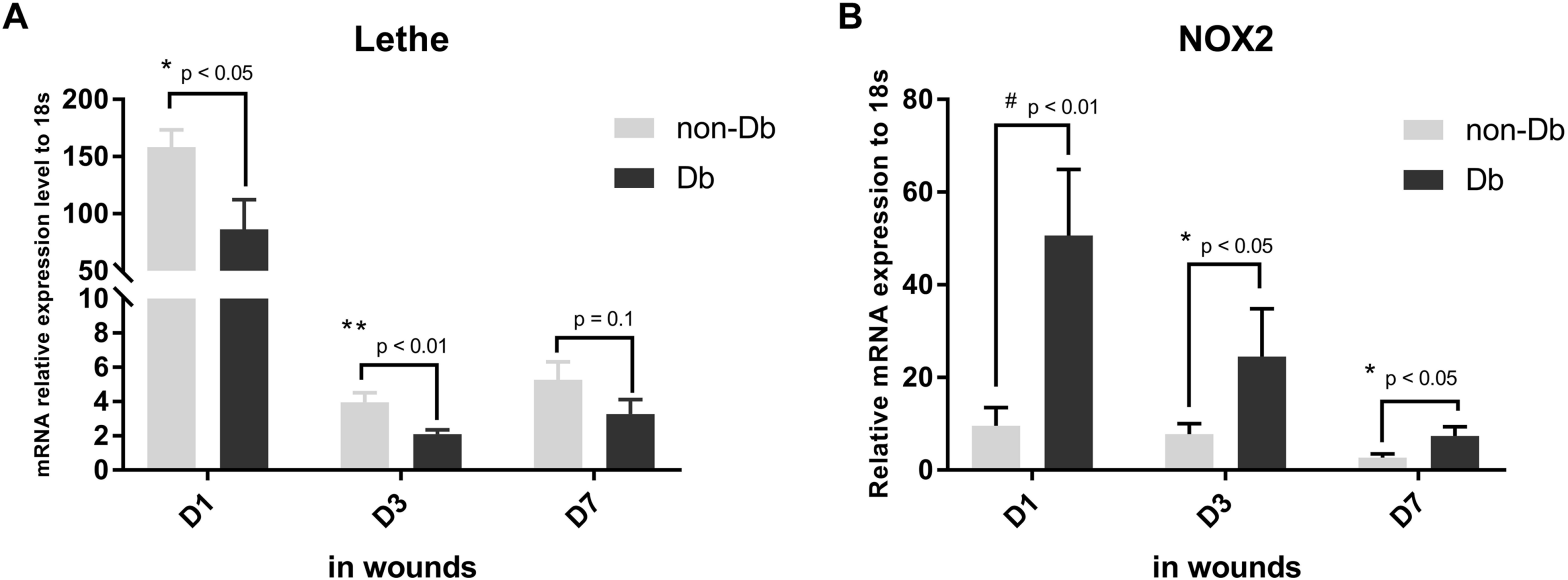
Lethe is down-regulated while NOX2 is up-regulated in diabetic wounds. mRNA analyses by RT-qPCR (mean ± SD, n = 5 per group) showed that Lethe is significantly downregulated in diabetic wounds 1, and 3 days after wounding (A), while NOX2 gene expression is significantly upregulated at the same time point (B), (mean ± SD, n = 5 per group).

Finally, we wanted to establish if these *in vitro* functions of Lethe were relevant in an *in vivo* model of impaired diabetic wound healing. 8 mm wounds were created on the back of Db/Db and Db/+ mice, harvested 1, 3 and 7 days post-wounding, and analyzed for Lethe and NOX2 gene expression. Diabetic wounds demonstrated significantly lower levels of Lethe gene expression 1, and 3 days post-wounding when compared to non-diabetic wounds (Fig 7A). This decrease in Lethe gene expression correlated with significantly higher expression of NOX2 gene expression in diabetic wounds (Fig 7B).

## Discussion

Diabetes is associated with dysregulation of nearly every step of the wound healing response. This dysregulation leads to the development of chronic wounds that contribute significantly to an impaired quality of life and an increased economic burden of disease for these patients. Given that the prevalence of diabetes is anticipated to increase in the US, as well as worldwide, developing effective wound care therapies remains of tremendous public health concern [25]. The role and mechanisms of ROS dysregulation in the wound healing response have been extensively examined. In the inflammatory phase of wound healing, inflammatory cells are activated and recruited to the wound area where they produce large amounts of ROS, matrix metalloproteinases, and pro-inflammatory cytokines [26, 27]. These inflammatory cells express high levels of NOX2 and produce high levels of ROS [10, 11] crucial to the neutralization of invading pathogens. However, excessive production of ROS leads to oxidative stress, which is a major contributor to the impaired healing of diabetic wounds [6, 7, 9]. Elevated and sustained ROS production can lead to persistent inflammation and continuous production of pro-inflammatory cytokines and matrix metalloproteinases, further impairing wound healing [28]. It is clear that a fine balance of ROS production is essential for effective wound healing [29, 30].

LncRNAs are non-coding RNAs greater than 200 nucleotides in length, which lack conservation across species and do not code for proteins [31–34]. While they can be found in either the nucleus or the cytoplasm, lncRNAs in the nucleus have the ability to recruit chromatin-modifying complexes to the DNA, block the binding of transcription factors to their promoters, or function as transcriptional co-activators [33, 35–37]. On the other hand, lncRNAs present in the cytoplasm regulate gene expression indirectly by sequestering and inhibiting microRNAs [38]. Lethe is a pseudogene lncRNA induced by IL-1b and TNF-a in mouse embryonic fibroblasts [20]. Lethe’s expression is dependent on NFkB activity; however, low levels of Lethe result in the upregulation of NFkB’s downstream targets, suggesting a negative feedback loop between Lethe and NFkB. It has been shown that Lethe binds to p65-NFkB in the nucleus, thereby preventing the activation of NFkB target genes such as IL-6 and IL-8 [20]. These studies support Lethe’s activity as an inhibitor of inflammation.

Here, we observed higher Lethe gene expression in diabetic BMM compared to non-diabetic BMM, confirming a baseline dysregulation of Lethe gene expression in diabetic BMMs relative to non-diabetic BMMs. We have also shown that exposing RAW macrophages to high glucose conditions increased the production of ROS. Furthermore, the increased production of ROS in RAW macrophages was attributable to increased expression of NOX2, a known producer of ROS. In addition, we found that high glucose decreased the expression of Lethe, an lncRNA involved in regulating NFkB, a key transcription factor in numerous inflammatory pathways. In order to determine if a connection exists between the dysregulation of Lethe and the increased production of ROS after high glucose treatment, we overexpressed Lethe in RAW macrophages, treated these cells with high glucose, and analyzed the expression of SOD2, SOD3, Catalase, and NOX2 and the production of ROS. Our results demonstrate that overexpression of Lethe specifically reduced the production of ROS. Lethe overexpression did not have any effect on the levels of SOD2, SOD3, or Catalase; however, Lethe overexpression significantly reduced NOX2 gene expression in RAW macrophages. These observations suggest that Lethe regulates oxidative stress in macrophages cultured under high glucose conditions through the regulation of NOX2 expression.

We then sought to define the mechanism by which Lethe regulates NOX2 expression and thereby ROS production. Previous studies have shown that the p65-NFkB complex can bind the promoter and induce the expression of NOX2 [24, 39]. Therefore, we measured the levels of p65-NFkB under low and high glucose conditions in RAW cells. Our data demonstrate that there was no difference in total p65-levels under these conditions. These results did not explain the higher expression of NOX2 that we observed, leading us to consider that the translocation of p65-NFkB to the nucleus may somehow be affected. Western blot analysis demonstrated that under high glucose conditions, higher levels of p65-NFkB were present in the nucleus of RAW cells. An upregulation in p65-NFkB levels in the nucleus permits higher binding of NFkB to the NOX2 promoter, thereby enabling upregulation of its expression. More excitingly, the high nuclear levels of p65-NFkB correlate with decreased expression of Lethe, consistent with previous reports suggesting that Lethe binds to NFkB. Thus, under high glucose conditions, Lethe’s expression is reduced, resulting in increased availability of free p65-NFkB to translocate to the nucleus and induce higher NOX2 expression.

In summary, this is the first study to describe the role of an lncRNA in diabetic wounds. We provide the first evidence that down-regulation of Lethe in diabetic wounds may play a role in the increased ROS production in diabetic wounds through the modulation of NOX2 expression. Future studies are warranted to determine if overexpression of Lethe *in vivo* would enhance the healing of diabetic wounds.
